# Racial Disparities in Lung Cancer Screening Among Veterans, 2013 to 2021

**DOI:** 10.1001/jamanetworkopen.2023.18795

**Published:** 2023-06-16

**Authors:** Neelima Navuluri, Samantha Morrison, Cynthia L. Green, Sandra L. Woolson, Isaretta L. Riley, Christopher E. Cox, Leah L. Zullig, Scott Shofer

**Affiliations:** 1 Division of Pulmonary, Allergy, and Critical Care, Department of Medicine, Duke University School of Medicine, Durham, North Carolina; 2 Durham Veterans Affairs Medical Center, Durham, North Carolina; 3 Duke Global Health Institute, Duke University, Durham, North Carolina; 4 Department of Biostatistics and Bioinformatics, Duke University School of Medicine, Durham, North Carolina; 5 Department of Population Health Sciences, Duke University, Durham, North Carolina

## Abstract

**Importance:**

Racial disparities in lung cancer screening (LCS) are often ascribed to barriers such as cost, insurance status, access to care, and transportation. Because these barriers are minimized within the Veterans Affairs system, there is a question of whether similar racial disparities exist within a Veterans Affairs health care system in North Carolina.

**Objectives:**

To examine whether racial disparities in completing LCS after referral exist at the Durham Veterans Affairs Health Care System (DVAHCS) and, if so, what factors are associated with screening completion.

**Design, Setting, and Participants:**

This cross-sectional study assessed veterans referred to LCS between July 1, 2013, and August 31, 2021, at the DVAHCS. All included veterans self-identified as White or Black and met the US Preventive Services Task Force eligibility criteria as of January 1, 2021. Participants who died within 15 months of consultation or who were screened before consultation were excluded.

**Exposures:**

Self-reported race.

**Main Outcomes and Measures:**

Screening completion was defined as completing computed tomography for LCS. The associations among screening completion, race, and demographic and socioeconomic risk factors were assessed using logistic regression models.

**Results:**

A total of 4562 veterans (mean [SD] age, 65.4 [5.7] years; 4296 [94.2%] male; 1766 [38.7%] Black and 2796 [61.3%] White) were referred for LCS. Of all veterans referred, 1692 (37.1%) ultimately completed screening; 2707 (59.3%) never connected with the LCS program after referral and an informational mailer or telephone call, indicating a critical point in the LCS process. Screening rates were substantially lower among Black compared with White veterans (538 [30.5%] vs 1154 [41.3%]), with Black veterans having 0.66 times lower odds (95% CI, 0.54-0.80) of screening completion after adjusting for demographic and socioeconomic factors.

**Conclusions and Relevance:**

This cross-sectional study found that after referral for initial LCS via a centralized program, Black veterans had 34% lower odds of LCS screening completion compared with White veterans, a disparity that persisted even after accounting for numerous demographic and socioeconomic factors. A critical point in the screening process was when veterans must connect with the screening program after referral. These findings may be used to design, implement, and evaluate interventions to improve LCS rates among Black veterans.

## Key Points

**Question:**

Are there racial disparities in initial lung cancer screening rates among veterans referred for screening after adjusting for various demographic and socioeconomic risk factors?

**Findings:**

In this cross-sectional study of 4562 veterans eligible for and referred for lung cancer screening at the Durham Veterans Affairs Health Care System, Black veterans had significantly lower odds of completing lung cancer screening even after adjusting for various demographic and socioeconomic risk factors.

**Meaning:**

These findings suggest that Black veterans have lower rates of lung cancer screening that are not fully explained by demographic and socioeconomic variables, underscoring the need for further qualitative studies on barriers to lung cancer screening as well as evidence-based interventions targeted to Black veterans.

## Teaser

This cross-sectional study of veterans referred for lung cancer screening examines whether racial disparities in screening exist within the Durham Veterans Affairs Health Care System.

## Introduction

Two large randomized clinical trials^1,2^ found that annual low-dose computed tomography (LDCT) for lung cancer screening (LCS) allows for early detection of lung cancer and decreases mortality by nearly 25%. These trials led to the endorsement of LCS by the US Preventive Services Task Force (USPSTF) in 2013, with a recent liberalization of screening eligibility in 2021.^3,4^

Similar to other cancer screening modalities, there are significant racial disparities in the application and use of LCS. Small studies^5,6,7,8^ have found that Black patients are less likely to have undergone LCS than White patients, despite evidence that Black males have the highest age-adjusted rates of lung cancer incidence and the highest lung cancer mortality among all US racial and ethnic groups. In addition, Black individuals are more likely to develop cancer at an earlier age and present with advanced-stage disease.^7,8^ These disparities persist in both smokers and never-smokers.^6,9^ Often-cited reasons for disparities in LCS rates include insurance status, rurality, environmental or occupational exposures outside of tobacco use, access to care and transportation, stigma, and challenges with patient-practitioner communication and shared decision-making.^6,10,11,12,13^

The Durham Veterans Affairs Health Care System (DVAHCS) implemented a centralized LCS program in 2013 as part of a national implementation program.^14^ Data from the early implementation period showed that 49.6% of eligible veterans across 8 different sites received a screening CT scan; stratification by race was not provided in this study.^14^ This finding compares with the estimated LCS rates of 5% to 17% nationally.^15,16,17^ Because several factors thought to be barriers to LCS, such as insurance status, screening cost, access to care, and transportation, are minimized within the Veterans Affairs (VA) system, we sought to further examine racial disparities in LCS within the DVAHCS. Our objectives were to examine whether racial disparities in LCS exist within the DVAHCS population, critical points in the LCS process that affect screening completion, and potential risk factors for not screening, with a specific focus on risk factors among Black veterans.

## Methods

### Study Design and Population

This retrospective cross-sectional study used the DVAHCS Lung Cancer Screening Database, which includes all veterans referred for LCS at the Durham VA since 2013 and data on their race, ethnicity, sex, smoking history, family history, decisions regarding screening, and CT scan results. The lung cancer screening process at the Durham VA is detailed in the eFigure in Supplement 1. The study was approved by the DVAHCS Institutional Review Board and received a waiver of consent determination because the data were deidentified. This study followed the Strengthening the Reporting of Observational Studies in Epidemiology (STROBE) reporting guidelines.

For this analysis, we included all veterans who were referred for LCS at the DVAHCS between July 1, 2013, and August 31, 2021, and self-identified as Black or White. We limited analyses to Black and White veterans given the small numbers of veterans in other racial groups and did not include ethnicity in the definition of self-identified race. We excluded veterans who died within 15 months of consultation placement, who were referred for LCS but ultimately did not meet the USPSTF criteria, or who had received a scan before referral (353 of 5499 [6.4%]). We used structured query language of the VA Corporate Data Warehouse to collect additional socioeconomic and comorbidity data, including percent-service connection, financial means, copayment, home zip code, and mental health comorbidities.

### Measures

Patient demographic characteristics, diagnosis codes, and family histories relevant to the study were collected from the database and the Corporate Data Warehouse. The index encounter was defined as the date of first LCS consultation placement. Specific details about individual measures can be found in the eMethods in Supplement 1.

### Outcomes

Our primary outcome of interest was LCS screening completion, which we defined as an individual having undergone chest CT scan after being referred for LCS. Screening performed outside the VA system is captured in our database for veterans who reported screening to program staff or for whom CT scan reports or images were shared. The percentage of patients eligible for LCS who (1) connected with the screening nurse to discuss LCS, (2) were scheduled for CT scan for LCS, and (3) received an LDCT were summarized, as was the time between LCS referral and CT scan for LCS completion.

### Statistical Analysis

Demographic and socioeconomic data by race are presented as mean (SD) or median (IQR) for continuous variables and numbers (percentages) for categorical variables. To examine whether screening completion was associated with race, we fit both an unadjusted logistic regression model with race and a multivariable logistic regression model with race and the demographic and socioeconomic factors of interest, including age at consultation, pack-year history, 1-year Care Assessment Need (CAN) score, current Nosos (Nosos-c) score, mental health, minimum distance to scanner, categories of rural-urban commuting area codes, service connection (percent measure of injury or illness that was caused by, or got worse because of, active military service), employment status, combat veteran status, marital status, and current smoking status (eMethods in Supplement 1). The CAN scores use VA electronic health record data to reflect the likelihood of hospitalization or death in an individual veteran patient compared with other patients at both 90 days and 1 year.^18,19^ Nosos scores are a measure of estimated cost a Veteran patient may have and use current year data to predict the current and prospective year risk scores.^20^ Scores are centered around 1, which indicates the national mean cost for veteran patients. Ninety-day CAN and prospective Nosos scores were omitted from these analyses because of collinearity. The multivariable logistic regression model included a significant interaction term between age categories and race; however, for comparability, the main effects of race and age were estimated by least-squares mean difference unadjusted odds ratios (ORs) or adjusted odds ratios (aORs) and presented with 95% CIs.

To examine whether time between referral and screening were associated with the screen completion rate by race such that varying follow-up times would be concerning (eg, survival analysis methods necessary), we conducted a sensitivity analysis for the primary outcome testing for an association between screening completion within 180 days and race for those with at least 180 days of follow-up (eMethods in Supplement 1). To examine which factors may be of most importance to Black veterans specifically, we conducted a subgroup analysis to examine the association between screening completion and these same factors within Black veterans. The multivariable model was chosen using a backward selection procedure based on the Akaike information criterion to examine which of the risk factors were most important.

For all models, linearity assumptions were assessed and relevant interaction terms between race and the demographic and socioeconomic variables were considered. Because of violations of linearity, the Nosos-c score was log-transformed and a spline plot for age at consultation was visualized. On the basis of the spline plot, age was categorized as younger than 60, 60 to 65, 65 to 70, 70 to 75, and older than 75 years. Given minimal missing data, models only used complete cases (N = 4331 in the full cohort and 1674 in the Black veterans subgroup).

All analyses were performed with SAS software, version 9.4 (SAS Institute Inc) and R, version 4.2.0 (R Foundation for Statistical Computing). A 2-sided *P* < .05 was considered statistically significant.

## Results

**Figure 1.**
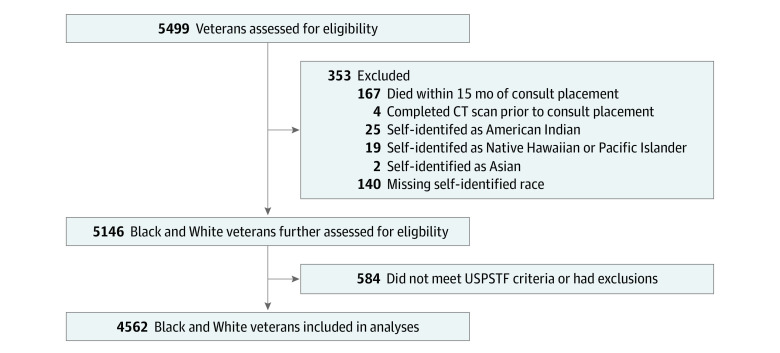
Participant Flow Diagram CT indicates computed tomography; USPSTF, US Preventive Services Task Force.

The cohort included 5499 veterans referred for LCS between July 2013 and August 2021, of whom 4562 Black and White veterans (mean [SD] age, 65.4 [5.7] years; 4296 [94.2%] male and 266 [5.8%] female; 1766 [38.7%] Black and 2796 [61.3%] White) met the USPSTF criteria for screening and our study inclusion and exclusion criteria (Figure 1 and Table 1). A total of 2970 of 4536 veterans (65.5%) were current smokers at the time of referral to LCS with a higher proportion of Black veterans currently smoking (1258 of 1757 [71.6%] vs 1712 of 2779 [61.6%]). However, Black veterans had a lower median (IQR) pack-year history (40 [35-50] vs 47 [40-60]). A total of 399 veterans (8.7%) served in a combat role (Table 1).

**Table 1.** Patient Characteristics by Race Among Veterans Referred for Lung Cancer Screening at the Durham Veterans Affairs Health Care System Who Met the US Preventive Services Task Force Eligibility Criteria, 2013-2021^a^

Characteristic	Total (N = 4562)	Black (n = 1766)	White (n = 2796)
Age at consultation, y			
Missing	3	1	2
Mean (SD)	65.4 (5.7)	64.5 (5.3)	66.0 (5.8)
Age at consultation categories, y			
Missing	3	1	2
<60	911 (20.0)	394 (22.3)	517 (18.5)
60-65	1247 (27.4)	594 (33.7)	653 (23.4)
65-70	1361 (29.9)	487 (27.6)	874 (31.3)
70-75	824 (18.1)	241 (13.7)	583 (20.9)
>75	216 (4.7)	49 (2.8)	167 (6.0)
Sex			
Male	4296 (94.2)	1674 (94.8)	2622 (93.8)
Female	266 (5.8)	92 (5.2)	174 (6.2)
Hispanic or Latino	28/4521 (0.6)	8/1752 (0.5)	20/2769 (0.7)
Missing	41	14	27
BMI			
Missing	9	4	5
Mean (SD)	29.0 (5.9)	28.5 (6.0)	29.3 (5.9)
Marital status			
Missing	24	5	19
Married	2195 (48.4)	703 (39.9)	1492 (53.7)
Never married	453 (10.0)	267 (15.2)	186 (6.7)
Separated or divorced	1672 (36.8)	720 (40.9)	952 (34.3)
Widowed	218 (4.8)	71 (4.0)	147 (5.3)
Currently smoking			
Total smokers	2970/4536 (65.5)	1258/1757 (71.6)	1712/2779 (61.6)
Missing	26	9	17
Pack-year history at consultation			
Missing	28	10	18
Mean (SD)	50.6 (22.1)	44.1 (15.1)	54.7 (24.7)
Median (IQR)	45.0 (38.0-55.0)	40.0 (35.0-50.0)	47.0 (40.0-60.0)
COPD diagnosis			
Total patients with COPD	216/4536 (4.8)	43/1757 (2.4)	173/2779 (6.2)
Missing	26	9	17
Personal history of cancer			
Total patients with history of cancer	263/4536 (5.8)	72/1757 (4.1)	191/2779 (6.9)
Missing	26	9	17
Family history of lung cancer			
Total patients with history of lung cancer	204/4536 (4.5)	45/1757 (2.6)	159/2779 (5.7)
Missing	26	9	17
Mental health comorbidity	2686 (58.9)	1159 (65.6)	1527 (54.6)
RUCA			
Missing	5	0	5
Highly rural	24 (0.5)	7 (0.4)	17 (0.6)
Rural	2537 (55.7)	793 (44.9)	1744 (62.5)
Urban	1996 (43.8)	966 (54.7)	1030 (36.9)
Distance to closest CT scanner, miles			
Missing	4	0	4
Mean (SD)	58.0 (162.5)	42.4 (113.2)	67.9 (186.5)
Median (IQR)	30.3 (20.3-44.1)	28.1 (15.7-40.3)	32.6 (23.1-48.6)
Educational level			
Missing	3894	1538	2356
Less than high school	52 (7.8)	29 (12.7)	23 (5.2)
High school graduate	208 (31.1)	76 (33.3)	132 (30.0)
Post–high school training	78 (11.7)	26 (11.4)	52 (11.8)
Some college	241 (36.1)	79 (34.6)	162 (36.8)
College graduate and/or postgraduate degree	89 (13.3)	18 (7.9)	71 (16.2)
Employment status			
Employed	1326 (29.1)	451 (25.5)	875 (31.3)
Not employed	2119 (46.4)	979 (55.4)	1140 (40.8)
Retired	663 (14.5)	194 (11.0)	469 (16.8)
Unknown	454 (10.0)	142 (8.0)	312 (11.2)
Service connection			
None	1721 (37.7)	567 (32.1)	1154 (41.3)
1%-49%	769 (16.9)	314 (17.8)	455 (16.3)
50%-100%	1900 (41.6)	799 (45.2)	1101 (39.4)
Other^b^	172 (3.8)	86 (4.9)	86 (3.1)
Combat veteran			
Yes	399 (8.7)	131 (7.4)	268 (9.6)
No	3188 (69.9)	1296 (73.4)	1892 (67.7)
Unknown	975 (21.4)	339 (19.2)	636 (22.7)
Period of service			
Korean period	56 (1.2)	8 (0.5)	48 (1.7)
Persian Gulf War	568 (12.5)	227 (12.9)	341 (12.2)
Vietnam period	3926 (86.1)	1525 (86.4)	2401 (85.9)
Other	12 (0.3)	6 (0.3)	6 (0.2)
CAN score at 1 y			
Missing	4	3	1
Mean (SD)	59.9 (24.0)	61.8 (23.0)	58.7 (24.6)
Median (IQR)	60.0 (40.0-80.0)	65.0 (45.0-80.0)	60.0 (40.0-80.0)
Nosos-c score			
Missing	176	75	101
Mean (SD)	1.3 (1.6)	1.4 (1.7)	1.2 (1.5)
Median (IQR)	0.8 (0.5-1.4)	0.8 (0.5-1.5)	0.7 (0.4-1.3)

Abbreviations: BMI, body mass index (calculated as weight in kilograms divided by height in meters squared); CAN, Care Assessment Need; COPD, chronic obstructive pulmonary disease; RUCA, rural-urban commuting area codes.

^a^ Data are presented as number/total number (percentage) of patients unless otherwise indicated.

^b^ Other includes aid and attendance, humanitarian emergency, Veterans Affairs pension, or Purple Heart recipient.

A greater proportion of Black veterans lived in an urban area as defined by rural-urban commuting area codes (966 [54.7%]), whereas a greater proportion of White veterans lived in a rural or highly rural area (1761 [63.1%]). This finding aligned with a shorter median (IQR) minimum distance to a VA CT scanner for Black vs White veterans (28.1 [15.7-40.3] vs 32.6 [23.1-48.6] miles). Most veterans were not employed (2119 [46.4%]) or were retired (663 [14.5%]) at the time of referral to LCS. A slightly higher proportion of Black (799 [45.2%]) compared with White (1101 [39.4%]) veterans had a VA service connection level of 50% or higher, which meant that they did not have to pay a copayment for a CT scan for LCS.

In terms of comorbidities, Black veterans had a slightly lower prevalence of chronic obstructive pulmonary disease compared with White veterans (43 of 1757 [2.4%] vs 173 of 2779 [6.2%]) but a higher prevalence of mental health or substance use diagnosis (1159 [65.6%] vs 1527 [54.6%]). The median (IQR) Nosos-c score was slightly higher among Black than White veterans (0.8 [0.5-1.5] vs 0.7 [0.4-1.3]) as was the CAN score at 1 year (65 [45-80] vs 60 [40-80]).

### Racial Disparities in Screening Completion

A total of 1692 veterans (37.1%) who were referred for LCS ultimately completed screening (Table 2). A total of 2707 veterans (59.3%) referred to LCS never connected with the LCS program despite a telephone call or informational mailer, indicating a critical point in the LCS process. The remaining veterans (1855 [40.7%]) either agreed to screening (1747 [38.3%]) or declined screening (108 [2.4%]), and 2870 (62.9%) veterans were ultimately not screened. The median (IQR) length of time between referral and CT scan request was 59 (48-84) days and between referral and CT scan completion was 65 (50-93) days (eTable 1 in Supplement 1).

**Table 2.** Rates of Connecting With Screening Nurse and Requesting and Completing Initial LDCT for Eligible Veterans^a^

Variable	Total (N = 4562)	Black (n = 1766)	White (n = 2796)
No contact with nurse	2707 (59.3)	1176 (66.6)	1531 (54.8)
Connected with nurse and veteran declined screening	108 (2.4)	25 (1.4)	83 (3.0)
Connected with nurse and veteran agreed to screening	1747 (38.3)	565 (32.0)	1182 (42.3)
LDCT requested by nurse	1745 (38.3)	565 (32.0)	1180 (42.2)
LDCT completed by veteran (screening complete)	1692 (37.1)	538 (30.5)	1154 (41.3)
Time between LCS referral and screening CT completion, median (IQR), d	65.0 (50.0-93.0)	64.0 (50.0-93.0)	65.0 (50.0-93.0)

Abbreviation: CT, computed tomography; LCS, lung cancer screening; LDCT, low-dose computed tomography.

^a^ Data are presented as number (percentage) of patients unless otherwise indicated.

Screening rates were lower among Black compared with White veterans (538 [30.5%] vs 1154 [41.3%]). Black veterans had 0.62-fold lower odds of screening completion in the unadjusted analysis (95% CI, 0.55-0.71). In an adjusted analysis accounting for age, smoking status, and pack-year history, Black veterans had a 0.72-fold lower odds of screening completion (95% CI, 0.59-0.87) (eTable 2 in Supplement 1) and a 0.66-fold lower odds of screening completion in an adjusted analysis accounting for those factors as well as other socioeconomic factors (95% CI, 0.54-0.80) (eTable 2 in Supplement 1).

The sensitivity analysis among the 4479 veterans (98.2%) who had at least 180 days of follow-up demonstrated that Black veterans had a 0.64-fold lower odds of screening completion within 180 days (95% CI, 0.56-0.73) (eTable 2 in Supplement 1), which agreed with the original analyses and indicated that follow-up time was not expected to have a large effect on the results.

Among the 1747 veterans who were able to connect with the screening nurse and agreed to screening, screening completion rates were high overall (1692 [96.9%]); however, they were still lower among Black compared with White veterans (538 [95.2%] vs 1154 [97.6%]), with Black veterans having 0.48-fold lower odds of screening (95% CI, 0.28-0.83) (eTable 3 in Supplement 1).

### Factors Associated With Screening Completion Among All Veterans

In the unadjusted model, veterans aged 70 to 75 years (OR, 0.65; 95% CI, 0.53-0.79) and those older than 75 years (OR, 0.53; 95% CI, 0.38-0.75) had lower odds of LCS completion compared with veterans younger than 60 years (eTable 4 in Supplement 1). Additionally, never having been married (OR, 0.73; 95% CI, 0.58-0.91) and current smoking (OR, 0.74; 95% CI, 0.65-0.84) were associated with significantly lower odds of screening completion in the total cohort. Veterans with a higher pack-year history (OR, 1.04 per 5 units; 95% CI, 1.02-1.05), service connection of 50% to 100% (OR, 1.39; 95% CI, 1.21-1.59), higher CAN score (OR, 1.04 per 5 units; 95% CI, 1.03-1.05), higher Nosos-c score (log transformed; OR, 1.21; 95% CI, 1.12-1.30), or combat veteran status (OR, 1.41; 95% CI, 1.14-1.74) were significantly more likely to complete LCS.

**Figure 2.**
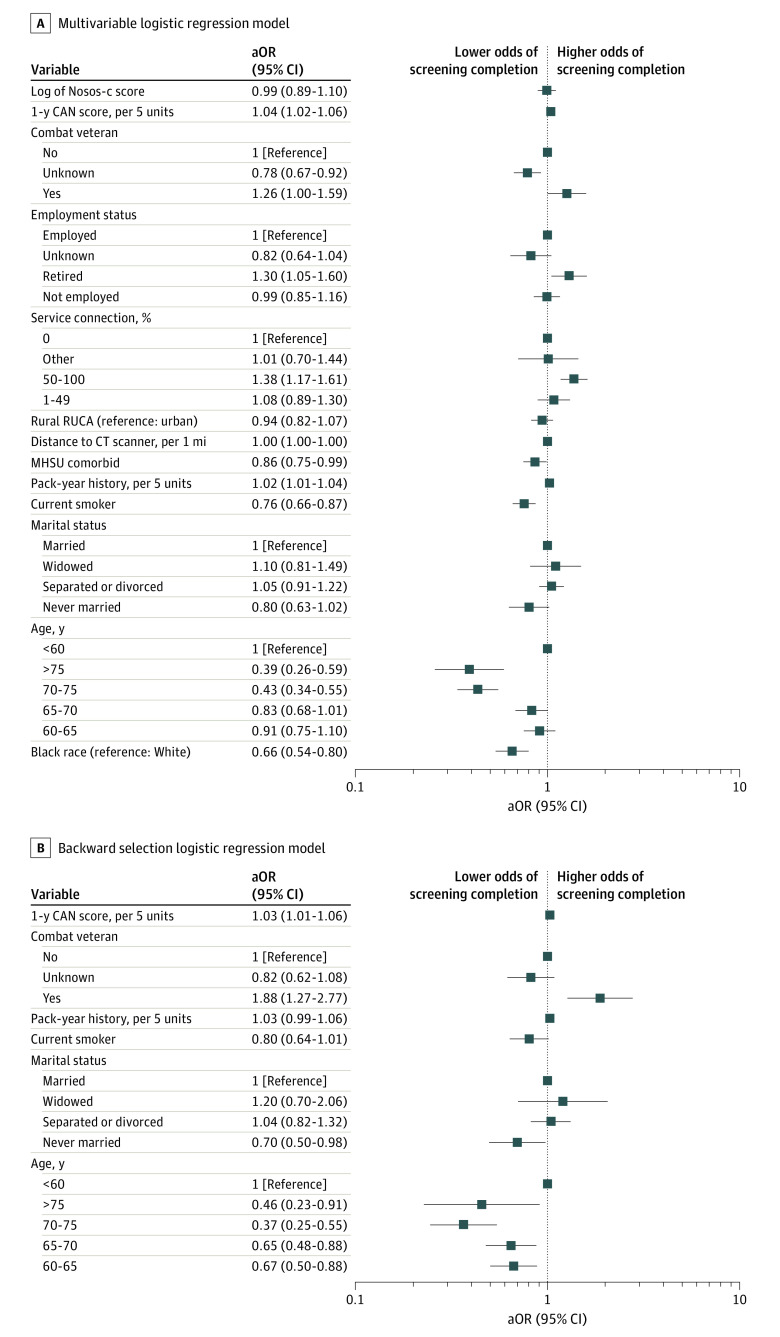
Association Between Screening Completion and Demographic and Socioeconomic Risk Factors A, Adjusted odds ratios (aORs) and 95% CIs from the multivariable logistic regression model (N = 4331). An interaction term was included between race and age, and the main effects are reported using ORs. B, aORs and 95% CIs from the backward selection logistic regression model for the association between screening completion and demographic and socioeconomic risk factors among Black veterans (N = 1674). CAN indicates Care Assessment Need; CT, computed tomography; MHSU, mental health or substance use diagnosis; Nosos-c, current Nosos; RUCA, rural-urban commuting area codes.

In the multivariable analysis (Figure 2A), mental health or substance use diagnosis was associated with lower odds of screening completion (aOR, 0.86; 95% CI, 0.75-0.99). Age at consultation category of 70 to 75 years (aOR, 0.43; 95% CI, 0.34-0.55) and older than 75 years (aOR, 0.39; 95% CI, 0.26-0.59) compared with younger than 60 years as well as current smoking status (aOR, 0.76; 95% CI, 0.66-0.87) were still associated with lower odds of screening completion. Marital status and Nosos-c score were no longer significant risk factors in the total cohort. A higher pack-year history (aOR, 1.02 for every 5 years; 95% CI, 1.01-1.04), service connection of 50% to 100% (aOR, 1.38; 95% CI, 1.17-1.61), combat veteran status (aOR, 1.26; 95% CI, 1.00-1.59), and a higher CAN score (aOR, 1.04 for every 5 units; 95% CI, 1.02-1.06) were still significantly associated with higher odds of screening completion after adjusting for the other covariates in the multivariable analysis. In addition, being retired vs employed was associated with higher odds of screening completion (aOR, 1.30; 95% CI, 1.05-1.60). For the multivariable model, an interaction term between age at consultation and race was included, and we found significantly lower odds of screening completion for Black compared with White veterans for age at consultation categories of 60 to 65, 65 to 70, and 70 to 75 years (eTables 5 and 6 in Supplement 1).

### Subgroup Analysis of Risk Factors for Screening Completion Among Black Veterans

To better understand factors associated with screening completion among Black veterans specifically, we examined the association between each factor and screening completion in the subgroup of Black veterans. Age at consultation categories of 60 to 65 (OR, 0.69; 95% CI, 0.52-0.91) and 70 to 75 years (OR, 0.46; 95% CI, 0.31-0.67) vs younger than 60 years were the only factors significantly associated with lower odds of screening completion in unadjusted analysis among Black veterans (eTable 7 in Supplement 1). In addition, having a service connection of 50% to 100% (OR, 1.40; 95% CI, 1.10-1.79), a combat veteran status (OR, 1.64; 95% CI, 1.13-2.38), a higher CAN score (OR, 1.03 for every 5-unit increase; 95% CI, 1.01-1.06), and a higher Nosos-c score (log-transformed; OR, 1.17; 95% CI, 1.03-1.32) were associated with significantly higher odds of screening completion. Notably, current smoking status, pack-year history, mental health comorbidity, and employment status were not significantly associated with screening completion among the subgroup of Black veterans.

Using multivariable backward selection, we found that age at consultation category, marital status, current smoking status, pack-year history, combat veteran status, and CAN score were the most important risk factors for screening completion among Black veterans (Figure 2B). All age groups vs younger than 60 years had lower odds of screening, with the group aged 70 to 75 years having the lowest odds of screening completion (aOR, 0.37; 95% CI, 0.25-0.55). Never being married was also associated with lower odds of screening completion (aOR, 0.70; 95% CI, 0.50-0.98). Black veterans who served in combat roles had approximately twice the odds of screening completion compared with those who did not serve in a combat role (aOR, 1.88; 95% CI, 1.27-2.77).

## Discussion

Black veterans had 34% lower odds of screening completion after referral for LCS in this cross-sectional study within the DVAHCS system, which uses a centralized screening process. This finding is consistent with several studies^5,17,21,22,23^ that found that Black individuals who met the USPSTF eligibility criteria were less likely to be screened for lung cancer. In fact, despite an overall screening rate that is much higher than in a study^17^ using Behavioral Risk Factor Surveillance System annual surveys (37.1% vs 17.0%), we report a similar racial disparity in LCS completion rates after accounting for various sociodemographic and clinical factors. This finding suggests that even though center characteristics, such as a centralized screening process or cost mitigation associated with a single-payer health system, may improve LCS rates overall, there are still racial disparities in LCS.

The critical point for not completing screening within this centralized system occurs when individual veterans must connect with the LCS program to be scheduled for a low-dose CT scan. Nearly 60% of veterans never connect with the program after they receive a telephone call or informational mailer. Among those who do make contact, few decline LCS and most veterans who agree to screening ultimately complete their CT scan.

To try to understand which factors may help explain racial disparities in LCS, we assessed a wide array of variables. Age at LCS referral was the only variable for which the association with screening completion differed significantly by race. When looking at the odds of screening completion by race in the total cohort as well as the subgroup of Black veterans, veterans in older age categories had lower odds of screening completion compared with patients younger than 60 years. Potential reasons for this finding may include personal factors regarding attitudes around screening or cancer diagnosis, as well as practitioner factors around utility of screening and life expectancy.^24^

Notably, the concordance index (c-index) for our multivariable models was 0.64 in the total cohort and 0.61 in the subgroup of Black veterans, indicating that we likely failed to capture information on several important variables in our models (eg, unmeasured confounders) despite the broad range of factors that were included. Key social determinants of health that were not captured in our analysis include educational level, health literacy, neighborhood-level data (eg, area deprivation index), and income. Other important variables likely include structural and systemic racism, implicit biases among clinicians, patient mistrust and skepticism, the racial and cultural environment of the health care setting, and differences in the quality of care among different racial groups.^25,26,27,28^ These factors negatively affect Black veterans and their health-related behaviors and outcomes but are difficult to measure or quantify, underscoring the need for further qualitative studies.

Current smoking status at LCS referral and mental health or substance use diagnosis were significantly associated with not screening in the total cohort multivariable analysis. This finding is consistent with findings that current smokers are less likely to undergo guideline-concordant breast, prostate, or colorectal cancer screening compared with former smokers or never-smokers and that individuals with mental illness had lower cancer screening rates across a variety of cancer types, despite increased cancer mortality rates.^29,30^ It also underscores the need for targeted interventions, such as tailored counseling as described by Flores et al,^31^ for individuals with mental illness or substance use.

Interestingly, higher pack-year smoking history was significantly associated with higher rates of screening completion, even after accounting for other covariates in the multivariable model. For every 5 pack-years, a veteran had a 2% increase in the odds of screening completion. This finding is concerning given evidence that Black individuals develop lung cancer at lower pack-year histories.^32,33^ The updated USPSTF guidelines are an important step in engaging individuals with a 20- to 30-pack-year history, but further community engagement and implementation work are necessary.

The costs of initial and annual low-dose CT scans for LCS are covered for veterans who are service connected by at least 50%. Those with lower service connection percentages were required to pay a $50 copayment for LCS during our study period.^34^ We found that a service connection level of 50% to 100% was unsurprisingly significantly associated with higher odds of screening completion compared with no service connection, even after accounting for other covariates. The VA recently announced that the cost of LCS would be covered for all patients regardless of percent service connection, which will be an important step in decreasing loss to follow-up.

Combat veterans also had significantly higher odds of screening completion, likely given their increased awareness and vigilance regarding potential carcinogenic exposures during their service. Finally, higher 1-year CAN scores were significantly associated with higher odds of screening completion, consistent with recent findings showing that individuals with worse health status have higher rates of LCS,^17^ likely because of a combination of factors, such as increased use of the health care system, higher interaction with practitioner teams, and less mistrust.

Commonly cited factors, such as distance from a scanner and rurality, were not significantly associated with screening completion in any of our analyses. Although rural residents in the general population are less likely to have an accredited screening facility close by,^10,11^ the effect of this variable on the veteran population is likely attenuated, highlighting the importance of well-connected referral networks and transportation assistance programs.

Among the subgroup of Black veterans, the most important risk factors for screening completion include age at consultation, marital status, current smoking status at referral, pack-year history, combat veteran status, and CAN scores. The results of the subgroup analysis allow us to focus on factors that can be used to help identify potential groups of Black veterans who may benefit from focused interventions to improve racial disparities in LCS rates.

### Strengths and Limitations

This study has both strengths and limitations. The main strength is that we were able to leverage data from the Corporate Data Warehouse to comprehensively examine LCS completion among a large cohort of patients referred for LCS since the implementation of the USPSTF guidelines. This allowed us to not only identify critical points for screening noncompletion but also gain a deeper understanding of the ways demographic and socioeconomic factors are associated with LCS. Limitations include that this is a single-center study among veterans, so our findings may not be generalizable to the general population; specifically, few women were in our cohort, restricting our assessment and analysis of risk factors among women and the interaction of race and sex. We also excluded 167 patients who died within 15 months of consultation placement. Although this was a relatively small number of patients, it may be of interest to incorporate these patients into future analyses using a survival framework. The retrospective nature of this study limits our ability to make definitive ascertainments of causal relationships. We also may be missing data on individuals who obtained LDCTs outside the VA system.

## Conclusions

In this cross-sectional study, Black veterans experienced significantly lower rates of screening completion after referral for initial LCS via a centralized program, even after accounting for numerous demographic and socioeconomic factors. The critical point for screening noncompletion in this process was in connecting with the screening program after a referral has been made. Key risk factors for lower odds of screening completion among Black veterans included age, marital status, and current smoking status, whereas pack-year history, combat veteran status, and care assessment needs were associated with higher odds of screening completion. These findings can inform qualitative and implementation research to design and assess interventions focused on improving LCS rates among Black veterans.

## Notes

Supplement 1. **eFigure.** Lung Cancer Screening Process at the Durham VA **eMethods.** Supplemental Methods **eTable 1.** Length of Time Between LCS Referral and CT Request or Completion Among Veterans Agreeing to LCS, by Race **eTable 2.** Association Between Race and Screening Completion Both Unadjusted and After Adjusting for Various Covariates **eTable 3.** Screening Completion by Race in the Subgroup of Patients Who Agreed to Screening (N = 1747) **eTable 4.** Univariable and Multivariable Logistic Regression Models for Associations Between Screening Completion and Factors (N = 4331) **eTable 5.** Separate Logistic Regression Models Testing if the Association Between Risk Factor and Screening Completion Differs by Race in the Cohort (N = 4562) **eTable 6.** Association Between Race and Screening Completion for Each Age Category From the Multivariable Model With Race and Age Interaction Term (N = 4331) **eTable 7.** Univariable and Multivariable Logistic Regression Models for Associations Between Screening Completion and Factors for Black Veterans (N = 1674)

Supplement 2.
Data Sharing Statement
