# Supplementary material for: Racial Disparities in Lung Cancer Screening Among Veterans, 2013 to 2021
Source: JAMA Netw Open. 2023 Jun 16;6(6):e2318795. doi: 10.1001/jamanetworkopen.2023.18795 (PMC10276308; doi:10.1001/jamanetworkopen.2023.18795)
Supplement: Supplement 1. — eFigure. Lung Cancer Screening Process at the Durham VA eMethods. Supplemental Methods eTable 1. Length of Time Between LCS Referral and CT Request or Completion Among Veterans Agreeing to LCS, by Race eTable 2. Association Between Race and Screening Completion Both Unadjusted and After Adjusting for Various Covariates eTable 3. Screening Completion by Race in the Subgroup of Patients Who Agreed to Screening (N = 1747) eTable 4. Univariable and Multivariable Logistic Regression Models for Associations Between Screening Completion and Factors (N = 4331) eTable 5. Separate Logistic Regression Models Testing if the Association Between Risk Factor and Screening Completion Differs by Race in the Cohort (N = 4562) eTable 6. Association Between Race and Screening Completion for Each Age Category From the Multivariable Model With Race and Age Interaction Term (N = 4331) eTable 7. Univariable and Multivariable Logistic Regression Models for Associations Between Screening Completion and Factors for Black Veterans (N = 1674) [file jamanetwopen-e2318795-s001.pdf]

Supplementary Online Content

Navuluri N, Morrison S, Green CL, et al. Racial disparities in lung cancer screening among veterans, 2013 to 2021. *JAMA Netw Open*. 2023;6(6):e2318795. doi:10.1001/jamanetworkopen.2023.18795

eFigure. Lung Cancer Screening Process at the Durham VA

eMethods. Supplemental Methods

eTable 1. Length of Time Between LCS Referral and CT Request or Completion Among Veterans Agreeing to LCS, by Race

eTable 2. Association Between Race and Screening Completion Both Unadjusted and After Adjusting for Various Covariates

eTable 3. Screening Completion by Race in the Subgroup of Patients Who Agreed to Screening (N=1747)

eTable 4. Univariable and Multivariable Logistic Regression Models for Associations Between Screening Completion and Factors (N=4331)

eTable 5. Separate Logistic Regression Models Testing if the Association Between Risk Factor and Screening Completion Differs by Race in the Cohort (N = 4562)

eTable 6. Association Between Race and Screening Completion for Each Age Category From the Multivariable Model With Race and Age Interaction Term (N = 4331)

eTable 7. Univariable and Multivariable Logistic Regression Models for Associations Between Screening Completion and Factors for Black Veterans (N = 1674)

This supplementary material has been provided by the authors to give readers additional information about their work.

© 2023 Navuluri N al. *JAMA Network Open.*

eFigure. Lung Cancer Screening Process at the Durham VA

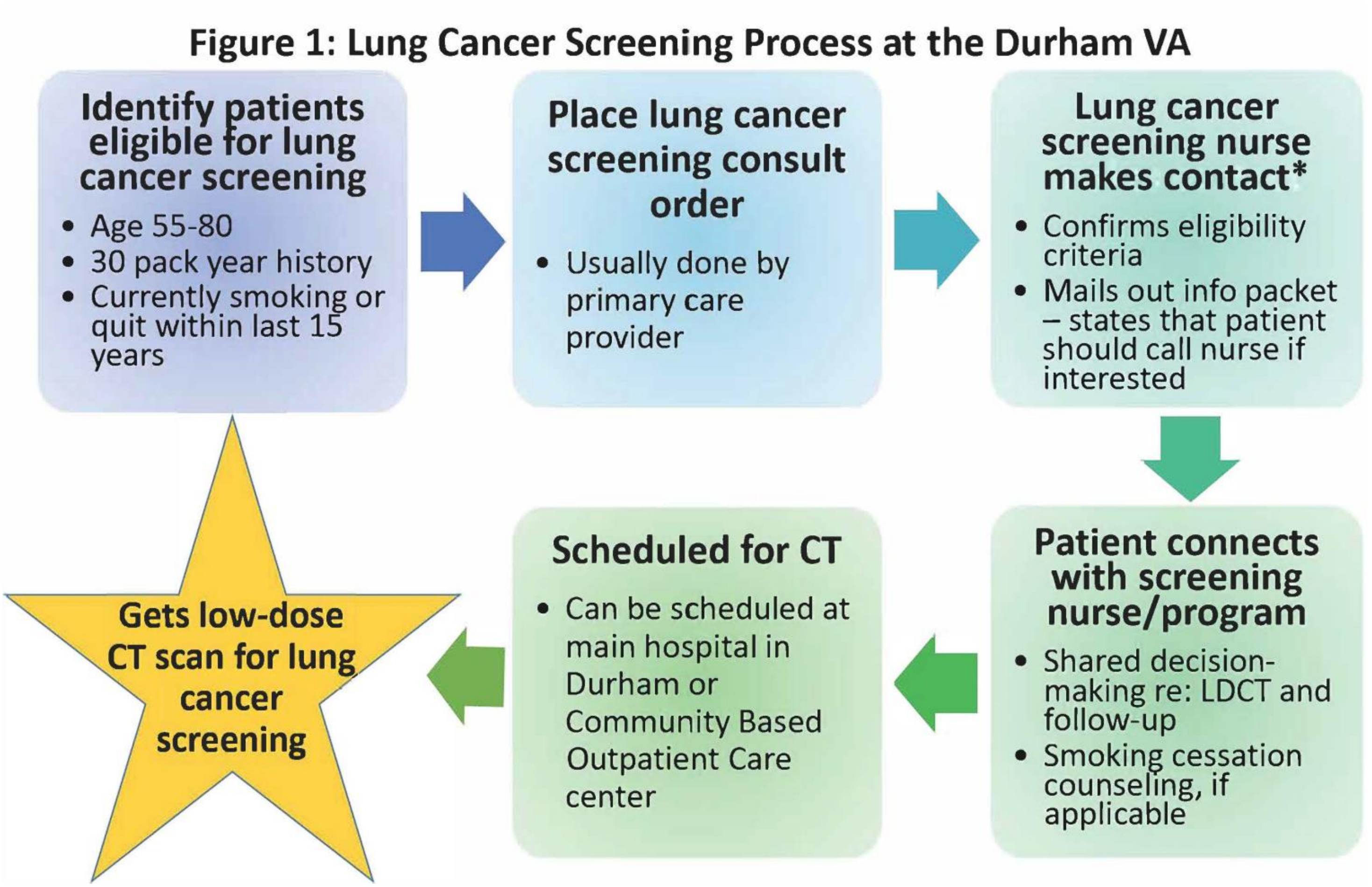

© 2023 Navuluri N al. *JAMA Network Open.*

eMethods. Supplemental Methods

Measures:

We retrieved the age, sex, race, body mass index (BMI), smoking history, diagnosis of COPD, personal diagnosis of cancer, family history of cancer, education level, employment status, marital status, means testing status, and service connection of each patient as documented at their index encounter. Smoking status was categorized as "former" and "current" and smoking pack-year history was included as a numerical value that was determined by the number of years smoked and average packs-per-day reported by the patient and recorded in the electronic medical system. Historical encounter and billing-associated diagnostic codes as classified using the Psychiatric Case Mix System (PsyCMS) International Classification of Diseases, Tenth Revision code were used to determine the presence of a mental health and substance use diagnosis.[1] Rurality was determined using the patient's zip code and classifying by Rural-Urban Commuting Area (RUCA) codes.[2] Cutoffs were applied to create a 3-category variable: urban, rural, and highly rural . Distance to CT scanner was determined by calculating the number of miles between the patient's address and the address of the closest scanner (Durham or Greenville VA). We also collected combat veteran status, Care Assessment Need (CAN) scores and Nosos scores from the CDW.

1. Wagner T, Gehlert E, Rosen A, Valenstein M. Updating the Psychiatric Case Mix System (PsyCMS) Mental Health and Substance Use Grouper for ICD-10-CM. Health Economics Resource Center 2016;

2. USDA ERS - Rural-Urban Commuting Area Codes [Internet]. [cited 2022 Oct 26];Available from: https://www.ers.usda.gov/data-products/rural-urban-commuting-area-codes.aspx

© 2023 Navuluri N al. *JAMA Network Open.*

eTable 1. Length of Time Between LCS Referral and CT Request or Completion Among Veterans Agreeing to LCS, by Race

	**Total**	**Black Veterans**	**White Veterans**
Days between LCS referral & CT Request, N	1745	565	1180
Mean (SD)	81.4 (75.4)	79.8 (65.0)	82.2 (79.9)
Median (Q1, Q3)	59.0 (48.0, 84.0)	58.0 (48.0, 84.0)	59.0 (47.5, 84.0)
Range	0-1416.0	0-561.0	0-1416.0
Days between LCS referral & CT Completion, N	1692	538	1154
Mean (SD)	88.4 (82.5)	86.5 (67.8)	89.3 (88.6)
Median (Q1, Q3)	65.0 (50.0, 93.0)	64.0 (50.0, 93.0)	65.0 (50.0, 93.0)
Range	0-1457.0	0-561.0	0-1457.0

© 2023 Navuluri N al. *JAMA Network Open.*

eTable 2. Association Between Race and Screening Completion Both Unadjusted and After Adjusting for Various Covariates

Model	Covariate	N	Odds Ratio (95% CI)	p-value	c-index
Full cohort (no missing data):					
Unadjusted	Black vs White	4562	0.62 (0.55 – 0.71)	<0.001	0.555
For comparison (complete case data only):					
Unadjusted	Black vs White	4331	0.62 (0.55 – 0.71)	<0.001	0.555
Adjusted for age, smoking status, and pack-year[a]	Black vs White	4331	0.72 (0.59 – 0.87)	<0.001	0.610
Adjusted for socioeconomic variables[b]	Black vs White	4331	0.66 (0.54 – 0.80)	<0.001	0.642
Sensitivity analysis, screening within 180 days[c]:					
Unadjusted	Black vs White	4479	0.64 (0.56 – 0.73)	<0.001	0.551

[a] After adjusting for age categories, pack-year history, and smoking status. An interaction term between race and age categories was included.

[b]After adjusting for age categories, pack-year history, 1-year CAN score, log-transformed Nosos-c score, mental health, minimum distance to scanner, RUCA categories, service connection, employment status, combat veteran, marital status, smoking status. An interaction term between race and age categories was included.

[c] For the sensitivity analysis, we assessed if there was association between race and screening within 180 days for patients with at least 180 days of follow up (N = 4479). Note: 1545 patients in this analysis were screened within 180 days and 2934 were not screened within 180 days.

© 2023 Navuluri N al. *JAMA Network Open.*

eTable 3. Screening Completion by Race in the Subgroup of Patients Who Agreed to Screening (N=1747)

LCS Screening Completed	Total (N=1747)	Black (N=565)	White (N=1182)	OR (95% CI)
No	55 (3.1%)	27 (4.8%)	28 (2.4%)	0.48 (0.28-0.83)
Yes	1692 (96.9%)	538 (95.2%)	1154 (97.6%)	

© 2023 Navuluri N al. *JAMA Network Open.*

eTable 4. Univariable and Multivariable Logistic Regression Models for Associations Between Screening Completion and Factors (N=4331)

	Univariable models	Multivariable model*
**Risk Factor**	**OR (95% CI)**	**aOR (95% CI)**
**Race**		
White	1 [Reference]	1 [Reference]
Black	**0.62 (0.55-0.71)**	**0.66 (0.54-0.80)**
**Age at consult categories**		
<60	1 [Reference]	1 [Reference]
60-65	0.93 (0.78-1.11)	0.91 (0.75-1.10)
65-70	1.09 (0.92-1.30)	0.83 (0.68-1.01)
70-75	**0.65 (0.53-0.79)**	**0.43 (0.34-0.55)**
>75	**0.53 (0.38-0.75)**	**0.39 (0.26-0.59)**
**Marital Status**		
Married	1 [Reference]	1 [Reference]
Never Married	**0.73 (0.58-0.91)**	0.80 (0.63-1.02)
Separated/Divorced	1.01 (0.89-1.16)	1.05 (0.91-1.22)
Widowed	1.04 (0.78-1.40)	1.10 (0.81-1.49)
**Currently Smoking**	**0.74 (0.65-0.84)**	**0.76 (0.66-0.87)**
**Pack Year History (5 units)**	**1.04 (1.02-1.05)**	**1.02 (1.01-1.04)**
**Mental Health or Substance Use Diagnosis**	1.02 (0.90-1.16)	**0.86 (0.75-0.99)**
**Distance to CT scanner (mi)**	1.00 (1.00-1.00)	1.00 (1.00-1.00)
**RUCA category**		
Urban	1 [Reference]	1 [Reference]
Rural	1.02 (0.90-1.16)	0.94 (0.82-1.07)
**Service connection**		
Not Service Connected	1 [Reference]	1 [Reference]
Service Connected 1-49%	1.01 (0.84-1.21)	1.08 (0.89-1.30)
Service Connected 50-100%	**1.39 (1.21-1.59)**	**1.38 (1.17-1.61)**
Other†	0.97 (0.69-1.36)	1.01 (0.70-1.44)
**Employment Status**		
Employed	1 [Reference]	1 [Reference]
Not Employed	1.01 (0.87-1.16)	0.99 (0.85-1.16)
Retired	1.19 (0.98-1.44)	**1.30 (1.05-1.60)**
Unknown	0.79 (0.62-1.00)	0.82 (0.64-1.04)
**Combat Veteran**		
No	1 [Reference]	1 [Reference]
Yes	**1.41 (1.14-1.74)**	**1.26 (1.00-1.59)**
Unknown	**0.80 (0.68-0.93)**	**0.78 (0.67-0.92)**
**CAN score 1 year (5 units)**	**1.04 (1.03-1.05)**	**1.04 (1.02-1.06)**
**Nosos-c score (log transformed)**	**1.21 (1.12-1.30)**	0.99 (0.89-1.10)

*The multivariable model included an interaction term between race and age (p-interaction=0.024) and had a c-index of 0.642. Univariable odds ratios (ORs) and multivariable adjusted odds ratios (aORs) are reported with 95% confidence intervals (CIs).

† Other = Aid and Attendance, Humanitarian Emergency, NSC VA pension, Purple Heart Recipient

© 2023 Navuluri N al. *JAMA Network Open.*

eTable 5. Separate Logistic Regression Models Testing if the Association Between Risk Factor and Screening Completion Differs by Race in the Cohort (N = 4562)

Risk factor	N	p-interaction*
Age at consult categories	4559	**0.010**
Age at consult (spline model)	4559	**<0.001**
Marital Status	4538	0.79
Current Smoker	4536	0.38
Pack Year History	4534	0.84
Mental Health Comorbidity	4562	0.56
Minimum distance	4558	0.71
RUCA category	4557	0.93
Service connection	4562	0.28
Employment Status	4562	≥0.99
Combat Veteran	4562	0.45
CAN score 1 year	4558	0.37
Log (Nosos-c)	4386	0.21

*Type 3 test statistic testing interaction term between risk factor and race (Black/White) These p-values were not adjusted for multiple testing.

The only difference by race between the assessed variables and screening completion was age at consult, which was significantly different between Black and White Veterans (p-interaction value < 0.001). The associations between marital status, current smoking status, pack-year history, mental health or substance use diagnosis, rurality, minimum distance to a CT scanner, service connection, employment status, combat veteran status, CAN score and Nosos scores and screening completion were not found to be significantly different by race in the cohort.

© 2023 Navuluri N al. *JAMA Network Open.*

eTable 6. Association Between Race and Screening Completion for Each Age Category From the Multivariable Model With Race and Age Interaction Term (N = 4331)

**Age category (years)**	**Odds Ratio (95% CI)**	**p-value**
Black vs White per category		
<60	0.87 (0.65 – 1.16)	0.35
60-65	0.51 (0.40 - 0.65)	<0.001
65-70	0.53 (0.42 – 0.68)	<0.001
70-75	0.54 (0.37 – 0.78)	0.001
>75	0.94 (0.45 – 1.99)	0.88

*Variables included in the model: race, age categories, pack-year history, 1-year CAN score, log-transformed Nosos-c score, mental health, minimum distance to scanner, RUCA categories, service connection, employment status, combat veteran, marital status, and smoking status. An interaction term between race and age categories was included.

© 2023 Navuluri N al. *JAMA Network Open.*

eTable 7. Univariable and Multivariable Logistic Regression Models for Associations Between Screening Completion and Factors for Black Veterans (N = 1674)

	Univariable models	Multivariable model
**Risk Factor**	**OR (95% CI)**	**aOR (95% CI)***
**Age at consult categories**		
<60	1 [Reference]	1 [Reference]
60-65	**0.69 (0.52-0.91)**	**0.67 (0.50-0.88)**
65-70	0.78 (0.58-1.04)	**0.65 (0.48-0.88)**
70-75	**0.46 (0.31-0.67)**	**0.37 (0.25-0.55)**
>75	0.62 (0.31-1.21)	**0.46 (0.23-0.91)**
**Marital Status**		
Married	1 [Reference]	1 [Reference]
Never Married	0.77 (0.56-1.07)	**0.70 (0.50-0.98)**
Separated/Divorced	1.11 (0.89-1.40)	1.04 (0.82-1.32)
Widowed	1.24 (0.73-2.10)	1.20 (0.70-2.06)
**Current Smoker**	0.83 (0.66-1.04)	0.80 (0.64-1.01)
**Pack Year History (5 units)**	1.02 (0.99-1.05)	1.03 (0.99-1.06)
**Mental Health Comorbidity (Yes vs No)**	0.99 (0.80-1.24)	--
**Minimum distance (mi)**	1.00 (1.00-1.00)	--
**RUCA category**		--
Urban	1 [Reference]	--
Rural	0.96 (0.78-1.18)	--
**Service connection**		--
Not Service Connected	1 [Reference]	--
Service Connected 1-49%	1.28 (0.94-1.75)	--
Service Connected 50-100%	**1.40 (1.10-1.79)**	--
Other†	1.10 (0.66-1.83)	--
**Employment Status**		--
Employed	1 [Reference]	--
Not Employed	1.08 (0.84-1.38)	--
Retired	1.17 (0.81-1.69)	--
Unknown	0.70 (0.44-1.09)	--
**Combat Veteran**		
No	1 [Reference]	1 [Reference]
Yes	**1.64 (1.13-2.38)**	**1.88 (1.27-2.77)**
Unknown	0.80 (0.61-1.05)	0.82 (0.62-1.08)
**CAN score 1 year (5 units)**	**1.03 (1.01-1.06)**	**1.03 (1.01-1.06)**
**Nosos-c score (log transformed)**	**1.17 (1.03-1.32)**	--

*Model chosen via backwards selection considering the 12 variables in the table (c-index 0.607). Data are presented using the unadjusted (OR) or adjusted odds ratio (aOR) with 95% confidence interval (CI).
† Other = Aid and Attendance, Humanitarian Emergency, NSC VA pension, Purple Heart Recipient

© 2023 Navuluri N al. *JAMA Network Open.*
