# Supplementary material for: Racial Disparities in Lung Cancer Screening Among Veterans, 2013 to 2021
Source: JAMA Netw Open. 2023 Jun 16;6(6):e2318795. doi: 10.1001/jamanetworkopen.2023.18795 (PMC10276308; doi:10.1001/jamanetworkopen.2023.18795)
Supplement: Supplement 2. — Data Sharing Statement [file jamanetwopen-e2318795-s002.pdf]

Data Sharing Statement

Navuluri. Racial Disparities in Lung Cancer Screening Among Veterans, 2013 to 2021. *JAMA Netw Open*. Published June 16, 2023. doi:10.1001/jamanetworkopen.2023.18795

Data
Data available: No

Additional Information
Explanation for why data not available: Department of Veterans Affairs (VA) places legal restrictions on access to VA data, which includes both identifying data and sensitive information. The analytic data sets used for this study are not permitted to leave the VA firewall without a Data Use Agreement, consistent with other studies based on VA data.
